# Equivalence of Conventionally-Derived and Parthenote-Derived Human Embryonic Stem Cells

**DOI:** 10.1371/journal.pone.0014499

**Published:** 2011-01-07

**Authors:** Julie V. Harness, Nikolay A. Turovets, Magdalene J. Seiler, Gabriel Nistor, Gulsah Altun, Larissa S. Agapova, David Ferguson, Louise C. Laurent, Jeanne F. Loring, Hans S. Keirstead

**Affiliations:** 1 Reeve-Irvine Research Center, Sue and Bill Gross Stem Cell Research Center, Department of Anatomy and Neurobiology, School of Medicine, University of California at Irvine, Irvine, California, United States of America; 2 International Stem Cell Corporation, Oceanside, California, United States of America; 3 Center for Regenerative Medicine, Scripps Research Institute, La Jolla, California, United States of America; Wellcome Trust Centre for Stem Cell Research, United Kingdom

## Abstract

**Background:**

As human embryonic stem cell (hESC) lines can be derived via multiple means, it is important to determine particular characteristics of individual lines that may dictate the applications to which they are best suited. The objective of this work was to determine points of equivalence and differences between conventionally-derived hESC and parthenote-derived hESC lines (phESC) in the undifferentiated state and during neural differentiation.

**Methodology/Principal Findings:**

hESC and phESC were exposed to the same expansion conditions and subsequent neural and retinal pigmented epithelium (RPE) differentiation protocols. Growth rates and gross morphology were recorded during expansion. RTPCR for developmentally relevant genes and global DNA methylation profiling were used to compare gene expression and epigenetic characteristics. Parthenote lines proliferated more slowly than conventional hESC lines and yielded lower quantities of less mature differentiated cells in a neural progenitor cell (NPC) differentiation protocol. However, the cell lines performed similarly in a RPE differentiation protocol. The DNA methylation analysis showed similar general profiles, but the two cell types differed in methylation of imprinted genes. There were no major differences in gene expression between the lines before differentiation, but when differentiated into NPCs, the two cell types differed in expression of extracellular matrix (ECM) genes.

**Conclusions/Significance:**

These data show that hESC and phESC are similar in the undifferentiated state, and both cell types are capable of differentiation along neural lineages. The differences between the cell types, in proliferation and extent of differentiation, may be linked, in part, to the observed differences in ECM synthesis and methylation of imprinted genes.

## Introduction

Human pluripotent stem cells (hPSCs) have tremendous potential for cell replacement, drug screening, predictive toxicology and developmental studies. There are multiple methods for generating hPSCs, including blastocyst derivation [1] derivation from single blastomeres [2], parthenogenesis [3], and induced pluripotency [4]. Pluripotent cell lines have also been derived from chromosomally abnormal spontaneous teratomas [5]. It is remarkable that these substantial differences in starting material and derivation methods yield cell lines that have fundamentally identical capabilities for pluripotency and self-renewal. However, the source of cell lines may have subtler consequences, perhaps leading to preferences for specific lineages or different tendencies toward tumorigenicity. Such phenotypic proclivities may be reflected in cellular morphology, behavior, or epigenetic state.

To begin to investigate differences among pluripotent cell types, we chose two cell types that are of great interest for development of cell replacement therapies, conventionally-derived hESCs, and hES cell lines derived from parthenote embryos (phESC). While conventionally-derived hESC lines comply with most of the proposed standards for use in clinical cell replacement therapies, there remains considerable concern about rejection of the transplanted stem cell derivatives, which would require the use of life-long immunosuppression. A number of approaches have been proposed to circumvent the need for immunosuppression after cell transplants. These methods include genetic modifications of cells; for example, replacing MHC genes, expression of Fas ligand, or modifications of other antigenic epitopes. Methods for making patient-specific pluripotent cells are promising, but they are currently impractical for clinical applications. Somatic cell nuclear transfer (SCNT) has not yet been achieved using human cells, and the most efficient methods for generating induced pluripotent stem cells (iPSCs) from an individual's somatic cells involve genetic engineering, which raises the bar for FDA approval considerably [4], [6].

The most viable short-term option for generation of histocompatible hPSCs is to create a cell bank of hESCs that are compatible with a wide range of HLA types. One means to increase the utility of such banks is to include parthenogenetic hESC lines, which may have simpler HLA haplotypes and therefore be histocompatible with a wider range of individuals. The first intentionally created phESCs were derived from a blastocyst inner cell mass obtained from an unfertilized oocyte activated by chemical stimuli [3]; these cells demonstrated typical characteristics displayed by hESC, including proliferation, expression of typical markers of pluripotency and differentiation *in vitro* and *in vivo* into cells of all germ layer lineages. Different activation techniques allow creation of either HLA heterozygous phESC that are totally HLA matched with oocyte donors, or HLA homozygous phESC which are histocompatible with significant segments of the human population [3], [7], [8], [9].

In order to determine whether parthenogenetic hESC lines and conventionally-derived hESC lines displayed any phenotypic differences that might affect their utility for human clinical applications, we compared hESC and phESC using a battery of cellular and molecular analyses. Our data revealed differences between hESC and phESC during propagation and differentiation, as well as molecular differences in imprinting and expression of ECM genes.

## Materials and Methods

### Undifferentiated Cell Culture

Eight cell lines were investigated, three conventionally-derived (H7, H9 and CSC14) and five parthenote-derived (LLC2P, LLC6P, LLC7P, LLC8P and LLC9P). The CSC14 line was provided by California Stem Cell Corporation. The five LLC lines were derived and described by International Stem Cell Corporation[3]; in that publication, LLC2P was referred to as phESC-1, LLC6P as phESC-3, LLC7P as phESC-4, LLC8P as phESC-5 and LLC9P as phESC-6.

Two different cell culture systems were used: feeder-free and on human neonatal skin derived fibroblasts (NSF) described in Revazova et al. [3]. In the feeder-free culture system, hESC and phESC cultures were passaged weekly with collagenase IV (Invitrogen) onto MatriGel™-coated flasks (Fisher, CB-40230) and conditioned growth medium was replaced every day. Growth medium consisted of KODMEM-F12 with 20% KOSR, 1% non-essential amino acids, 0.5% GlutaMAX-I (all from Life Sciences Inc.) and 7 µl/l β-mercaptoethanol (Sigma-Aldrich). This medium was supplemented with 4 ng/ml bFGF (GF003-AF, Millipore) and conditioned on irradiated mouse embryonic fibroblasts (MEFs) overnight, then filtered in a 0.1 µm PES filter (Fisher) before being supplemented with an additional10 ng/ml bFGF. The number of colonies were counted at Day 1 after passaging.

In the NSF feeder culture system, undifferentiated phESC and hESC were maintained on a feeder layer of mitomycin C mitotically inactivated NSF in KODMEM/F12 (Invitrogen) supplemented with 15% KOSR (Invitrogen), 0.05 mM non-essential amino acids (Invitrogen), 2 mM GlutaMAX-I (Invitrogen), penicillin/streptomycin (Invitrogen), 55 µM β-mercapthoethonol (Invitrogen) and 5 ng/ml recombinant human FGF-basic (PeproTech). Cultures were manually passaged at 1∶4 – 1∶6 split ratio every 5–7 days using collagenase IV (Invitrogen).

### PCR

Two different PCR arrays from SA Biosciences (Frederick, MD) were run on samples of four lines (CSC14, H7, LLC6P and LLC8P): the Stem Cell Array (PAHS-405, SA Biosciences) on undifferentiated samples and the ECM array (PAHS-013, SA Biosciences) on NPC. Cells were exposed to collagenase (in the case of undifferentiated samples) (Invitrogen) or TrpLE (in the case of NPC) (Invitrogen), pelleted and exposed to 1 ml of Trizol (15596-018, Invitrogen) per 1×10∧6 cells, then homogenized. The TURBO DNA-free Kit (AM1907, Applied Biosystems, Foster City, CA) was used and the manufacturer's protocol was followed. The RNeasy Mini Kit (74104, Qiagen, Valencia, CA) was used and RNA was purified according to the manufacturer's protocol. The SA Biosciences First Strand Kit (C-03) was used, followed by the SA Biosciences RNA QC PCR Array (PAHS-999). Three Stem Cell Array plates were run for each undifferentiated sample and three ECM plates were run for each NPC sample, for a total of 12 plates of each type. Data was analyzed with the online SABiosciences webportal (http://www.sabiosciences.com/pcr/arrayanalysis.php).

### Methylation Microarray

Microarray-based DNA methylation analysis was performed on two technical replicates for each line assayed on the Illumina Infinium Human Methylation 27 BeadChip array (Illumina, Inc.). This array interrogates 27,578 CpG sites representing roughly 14,500 genes. Aliquots containing 1×10^6^ cells of H7, CSC14, LLC2P, LLC6P, LLC7P, LLC8P and LLC9P were flash frozen for DNA purification and subsequent methylation analysis. Genomic DNA was purified using the DNeasy Kit (Qiagen) and quantified with Picogreen (Invitrogen). These samples were compared to the hESC lines HES2, KSR-HSF6, KSR-Miz6 and Mel1; the primary cell lines NuFF's, Feeder-12age-HDF, HDF-neonatal, Huevec-BF4, SC01-C-glia, SC11-glia, SC30-C-fibroblasts, SC31-C-MSC; and tissue samples from colon, female Caucasian blood, ovary, prostate and breast. Five hundred nanograms of genomic DNA from technical replicates of each cell line were bisulfite-converted using the EZ DNA Methylation Kit (Zymo Research). The bisulfite-converted DNA was processed and hybridized to the Human Methylation 27 BeadChip (Illumina, Inc.), according to the manufacturer's protocol and scanned on a BeadArray Reader (Illumina, Inc.). The degree of methylation was expressed as the β-value, where β = methylated/(methylated+unmethylated). The β-values for each sample were normalized by range-scaling the data for each probe, using data from fully methylated (generated by treating genomic DNA with SssI DNA methyltransferase), fully unmethylated (generated by whole-genome amplification of genomic DNA), and partially methylated (generated by mixing equal amounts of fully methylated and fully unmethylated genomic DNA) control samples run in triplicate. Because CpG sites assigned to imprinted genes were not necessarily imprinted, it was necessary to devise a post hoc method of identifying imprinted loci. Therefore, probes assigned to imprinted genes were selected and a filter applied to select only sites that were partially methylated in tissue samples (as is expected of imprinted loci).

### NPC Differentiation

NPC differentiations were carried out on four lines: H7, CSC14, LLC6P and LLC8P. LLC6P and LLC8P were selected from the other phESC lines because in the undifferentiated state they most closely resembled conventionally-derived hESC in growth rate and colony morphology. Differentiations were carried out using each of the four lines at the same time with the same reagents and media. Differentiations were repeated with three successive passages of cells for a total of three grows. All four cell lines used for differentiation were brought to subconfluence in the feeder-free culture system before beginning the differentiation protocols.

H7 passage 38, 40 and 41 were used. CSC14 passage 39, 40 and 41 were used. LLC6P passage 38, 39 and 40 were used. LLC8P passage 31, 32 and 33 were used. In a novel differentiation protocol, subconfluent hESC were dissociated with collagenase IV and plated in ultra low adherence 6-well plates for sphere formation at a density of 200,000 cells/cm^2^. For the first two days, cells were fed 50% conditioned medium (described above) and 50% neuron medium (NM). NM consisted of DMEM-F12+GlutaMAX, 2% B27, 1% ITS (insulin, transferrin, selenium) (Invitrogen), 1 mM MgCl^2^ (M4880, Sigma) and 4 ng/ml bFGF (Chemicon). From day 3 forward, cells were fed 100% NM. On days 2–6, medium was further supplemented with 10 µM all-trans retinoic acid (RA) in DMSO (Sigma-Aldrich). Cells were fed daily until day 6, after which they were fed three days a week, typically Monday, Wednesday and Friday. On day 14, cells were plated onto Matrigel-coated flasks at a density of 200,000 cells/cm^2^. On day 21, cells were dissociated with TrypLE and seeded onto laminin-coated (11243217001, Roche, Palo Alto, CA) coverslips in 12-well plates (07-200-82, Fisher) at a density of 40,000 cells/cm^2^. On day 24, cells were fixed for 15 minutes in 4% paraformaldehyde (PFA) (15714-S, Electron Microscopy Sciences, Hatfield, PA) for immunocytochemistry (ICC). On the day of plating for ICC, cells were exposed to TrpLE for 3 minutes, suspended at lower concentration in HBSS (Invitrogen), gently triturated with a 25 ml pipet 7 times and centrifuged at 250 g for three minutes. The resulting pellet was resuspended in HBSS once more and samples were taken for cell counts. Cells were counted on a hemocytometer until at least 100 cells of each sample were counted.

### RPE Differentiation

RPE differentiation was conducted using 2 distinct protocols, both of which did not require a sphere-forming stage. The first protocol was used to illustrate the principle that phESC were capable of end-product differentiation using protocols that lack a sphere-forming stage. The second protocol was used to confirm this finding.

In the first method, RPE differentiation as described by Klimanskaya et al. [10] was carried out on four lines: H9, LLC2P, LLC6P and LLC8P. Briefly, undifferentiated hESC and phESC, adherent on a feeder layer of human neonatal skin fibroblasts, were allowed to overgrow (about 7–10 days after passaging) until the colonies lost their tight borders and became multilayered, at which time bFGF was withdrawn from culture medium, inducing spontaneous differentiation. Conditions were maintained for 4–8 weeks during which time the medium was changed every 1–2 days. To isolate colonies of RPE- like cells, adherent cultures of spontaneously differentiated hESC and phESC cells were rinsed with PBS twice and incubated in collagenase IV at 37°C until the monolayer loosened. Cells from pigmented regions were scraped off with a glass capillary, washed in fresh culture medium and plated onto gelatin-coated plates in RPE medium (high glucose KODMEM supplemented with 500 µg/mL of penicillin, 500 µg/mL of streptomycin, 1% non-essential amino acids, 2 mM of GlutaMAX-I, 0.1 mM_*β*-mercaptoethanol, 7% SR, and 5% fetal bovine serum (all Invitrogen)). The medium was changed after the cells attached (usually in 1–2 days) and every 5–7 days after that; the cells were passaged every 2–4 weeks with 0.05% Trypsin/0.53 mM of EDTA (Invitrogen).

In the second method, a novel protocol was followed. Undifferentiated hESC and phESC growing in matrigel-adherent culture were brought to confluence prior to differentiation. On days 1 and 2 of differentiation, cells were fed serum replacement (SR) medium supplemented with 10 ng/ml bFGF. After dissociation, cells were incubated for 3 days in SR medium supplemented with 10 ng/ml bFGF and 10 µM RA. SR medium consisted of DMEM/F12 with 15% knockout serum replacement (Invitrogen). For the duration of the protocol, cells were fed RPE medium supplemented with 5 ng/ml bFGF and 10 µM RA. RPE medium consisted of DMEM/F12 with B27 supplement (Invitrogen) and Insulin/Transferrin/Selenium supplement (Invitrogen) as per manufacturer's recommendation. On day 20, cells were plated onto a Matrigel-coated adherent substrate. On day 42 of the differentiation protocol, pigmented clusters of cells were mechanically isolated and plated in uncoated flasks in RPE medium.

### Immunocytochemistry

In some cultures of undifferentiated hESC and phESC the mitotic index was determined using BrDU incorporation assays and Ki67 immunostaining. Undifferentiated phESC were plated in 4-well chamber slides 3 days before hESC were plated to ensure that both populations would reach 60% confluence on the same day. At the point of 60% confluence, media in BrdU wells was supplemented with 10 µM BrdU (Sigma-Aldrich). Twenty-four hours later, chambers were rinsed three times with warm HBSS and fixed in 4% paraformaldehyde (Electron Microscopy Sciences) for 15 minutes. Immunocytochemistry was carried out as described in the following paragraph regarding NPC cultures except the primary antisera used (mouse anti-BrdU, 1∶250, Millipore; rabbit anti-Ki67, 1∶1000, Abcam).

NPC cultures were washed in PBS and fixed in 4% paraformaldehyde (Electron Microscopy Sciences) for 15 minutes, then washed three times in PBS. Immunocytochemical staining was performed using standard protocols. Slides were blocked for 30 minutes at room temperature in 5% normal goat serum (NGS) (Invitrogen). Primary antisera (mouse anti-Nestin, 1∶200, Chemicon, Temecula, CA; rabbit anti-Pax 6, 1∶500, Chemicon; mouse anti-Map2, 1∶500, Chemicon; rabbit anti-Olig1, 1∶200, Chemicon; mouse anti-SSEA, 3/4 1∶200, Chemicon; rabbit anti-GFAP, 1∶1000, Chemicon) were diluted in 5% NGS and incubated overnight at 4°C. Slides were washed three times in PBS and incubated one hour at room temperature in AlexaFluor conjugated antisera (goat anti-mouse IgG (H+L) AlexaFluor 488, A11029, goat anti-rabbit IgG (H+L) AlexaFluor 488, A11034, Invitrogen). Slides were washed 3 times in ddH_2_O and incubated for 5 minutes at room temperature in 3.2 µM Hoechst (H3569, Invitrogen). Slides were washed 3 times in ddH_2_O and coverslipped using Fluoromount G (0100-01, Birmingham, AL). Images were captured using a Nikon Eclipse Ti microscope with 10X and 20X objectives and a Nikon Digital Sight DS-Fi1 camera.

RPE cultures were washed in PBS and fixed in 4% paraformaldehyde at room temperature for 15 minutes. Following permeabilization with 0.1% Triton-X100/PBS for 40 min, the cells were rinsed with PBS and incubated overnight at 4°C in a humidified chamber with the primary antibody: ZO1 (1∶50, Invitrogen), RPE-65 (1∶250, Novus Biologicals), Bestrophin (1∶25, Novus Biologicals), Occludin (1∶50, Santa Cruz Biotechnology, Inc.) and CRALBP (1∶50, Santa Cruz Biotechnology, Inc.). The cells were rinsed three times with PBS for 15 min and incubated at room temperature for 1 h with the secondary antibody: Alexa Fluor 546 donkey anti-mouse, Alexa Fluor 488 donkey anti-mouse, Alexa Fluor 546 donkey anti-rabbit or Alexa Fluor 488 donkey anti-goat (1∶1000, Invitrogen). The cells were then washed five to six times in PBS, covered with mounting medium with DAPI (Vector Laboratories, Inc.) and examined with the microscope (Zeiss Axio Imager).

### Western Analysis

The cells were lysed on ice in chilled RIPA lysis buffer (Millipore) and supplemented with protease inhibitor cocktails (Roche Applied Science). The cell extracts were centrifuged at 14,000 *g* for 15 minutes at 4°C. Supernatants were collected after centrifugation. Protein content was quantified using the Bradford assay (Sigma-Aldrich) according to the manufacturer's guidelines. The protein samples (50 µg per lane) were electrophoretically separated by SDS-PAGE in 4–20% gradient gel (PAGEgel, Inc.). After electrophoresis, proteins were transferred to a Hybond-P polyvinylidene difluoride (PVDF) membrane (GE Healthcare Biosciences). Membranes were blocked for 2 hours in 5% nonfat milk in PBS-0.05% Tween-20 and incubated 2 hours in 5% nonfat milk containing primary antibodies: RPE-65 (1∶5000, Novus Biologicals), Bestrophin (1∶1000, Novus Biologicals) or NCAM1 (1∶2000, Santa Cruz Biotechnology, Inc.). Membranes were washed in PBS-0.05% Tween-20, then incubated for 1 hour at room temperature with secondary HRP-conjugated antibody goat anti-mouse (1∶1000, Millipore). After the membranes were washed, the proteins were visualized using Enhanced Chemiluminescence Plus Western blotting detection system (GE Healthcare Biosciences) according to the manufacturer's protocol. Protein bands were detected by exposure to autoradiographic film (Amersham Hyperfilm ECL, GE Healthcare Biosciences).

### RPE Phagocytosis

RPE cultures generated from the LLC6P line were processed with the pH-rhodo BioParticles Conjugates for Phagocytosis kit (Invitrogen). Lyophylized pHrodo BioParticles fluorescent particles were dissolved in RPE medium at a concentration of 1 mg/mL according to manufacturer's recommendations. Adherent cultures in 6-well plates were fed with medium containing fluorescent particles. Beginning 5 minutes after addition of fluorescent particles, plates were imaged every 15 minutes for 2.5 hours with a Leica LSM 510 using 550 nm excitation and a 10x objective.

Cells were fixed with 4% paraformaldehyde after the assay. Small sheets were taken off the dish, placed on a slide, coverslipped with DAPI (4′6′-diamidino-2-phenylinode hydrochloride) containing Vectashield mounting medium (Vector Labs), and imaged on a Leica LSM710 confocal microscope, using a 63x objective to verify that the particles were inside the cells.

## Results

### Undifferentiated Cell Culture

**Figure 1:**
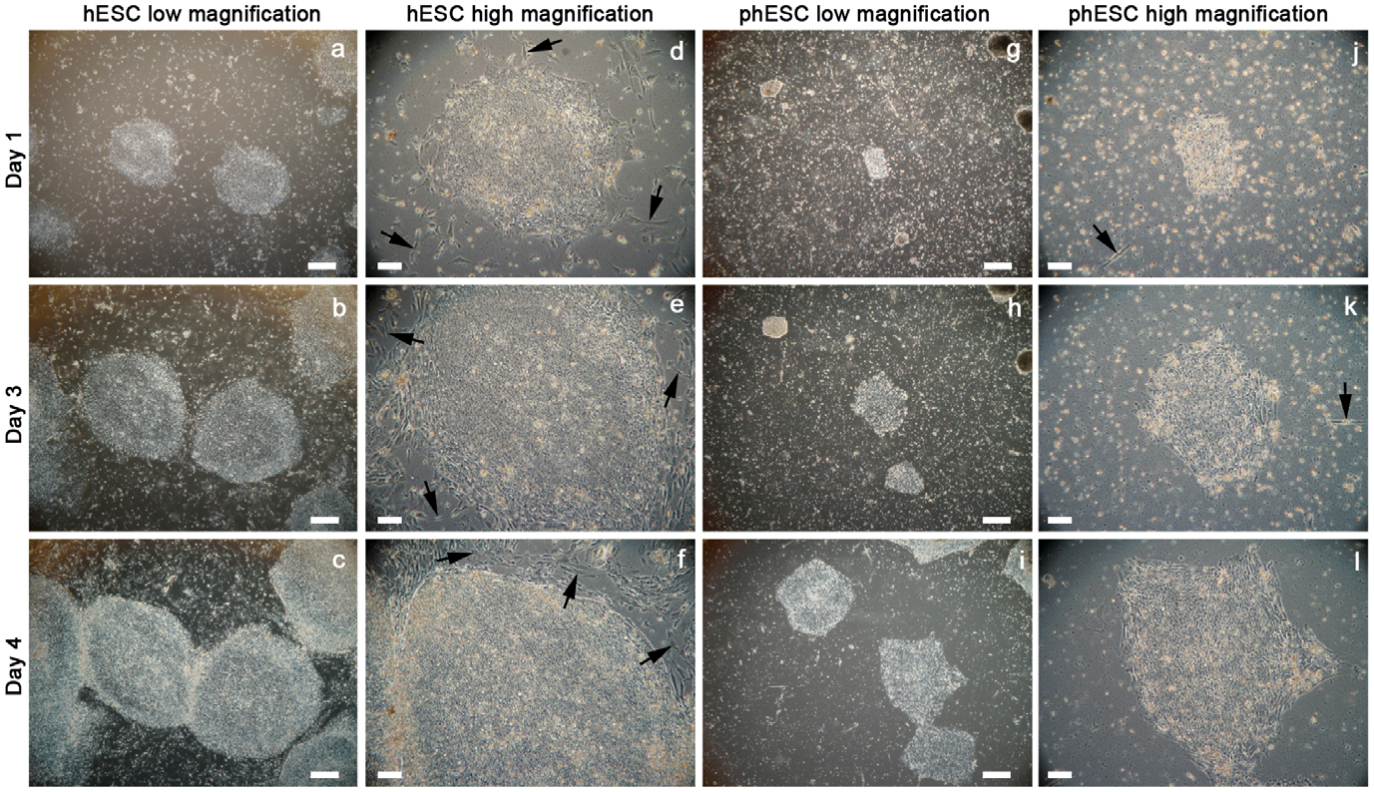
hESC and phESC differ morphologically. Conventionally-derived (a–f) and parthenote-derived (g–l) hESCs have distinct morphology and growth patterns in the undifferentiated state. High magnification and low magnification images have been provided for each colony to illustrate the slower growth of parthenote-derived colonies, irregular colony shape and lack of stromal cells (long, thin cells between colonies indicated by arrows) in the phESC cultures. Day 1 after plating (a, d, g and j). Day 2 after plating (b, e, h and k). Day 4 after plating (c, f, i and l). Scale bars represent 20 µm (a–c and g–i) and 10 µm (d–f and j–l).

In the undifferentiated state, phESC exhibited different growth characteristics than hESC. Grown in feeder-free conditions, hESC colonies were observed within 1 day of passaging and cultures reached confluence within 7 days. In the same conditions, phESC colonies were observed within 1 day and reached confluence in 10–12 days. Statistically fewer (p<0.01) phESC colonies (38 colonies +-8) adhered after passaging when cells were split at the same plating density (50,000 cells/cm^2^) as conventionally-derived hESC (202 colonies +-36). Once plated, colonies exhibited altered morphology (Fig. 1). The phESC colony-stroma border was irregular and not clearly defined. The hESC colonies were more round in shape and clearly delineated from surrounding stroma. Stroma, the long, thin cells between stem cell colonies, were not as prevalent in phESC cultures.

The rate of proliferation differed between hESC and phESC. BrdU incorporation assays indicated that the average number of BrdU positive cells was statistically greater (p<0.01) in hESC cultures (11.23%±1.44%) than phESC cultures (4.57%±1.07%). Ki67 staining indicated that the average number of Ki67 positive cells was not significantly different (p<0.01) between hESC cultures (97.92%±0.41%) and phESC cultures (98.45%±0.45%).

**Figure 2:**
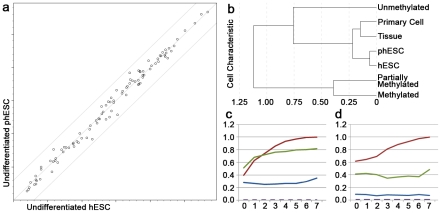
Undifferentiated colonies differed with respect to imprinted genes. PCR arrays did not reveal differences in the undifferentiated state (a). Parthenote-derived lines LLC6P and LLC8P are plotted on the y-axis and conventionally-derived lines H7 and CSC14 on the x-axis. Methylation microarray revealed differences between parthenote-derived lines and conventionally-derived lines, most notably in imprinted genes. Cluster analysis (b) indicated that phESC and hESC were closely related to each other and more methylated than tissue and primary cell lines. phESC and hESC are distinguishable when complete methylation data are included (c). phESC and hESC diverge further when methylation data include only imprinted genes (d). Methylation data, normalized with tissue lines as the unmethylated baseline (c and d). In c and d, correlation is plotted on the y-axis and level of discordance between 7 phESC lines is plotted on the x axis (e.g. 4 means 3 phESC lines were allowed discordance). Red represents phESC lines, green represents hESC lines, blue represents primary cell lines and dashed purple represents tissue lines.

Gene expression as determined by pluripotency-specific PCR array showed no differences between undifferentiated samples of hESC and phESC (Fig. 2a). Methylation microarray (GEO Series accession number GSE25538) revealed that epigenetically, phESC and hESC were more similar to each other than they were to cell line and tissue samples, but they were not indistinguishable (Fig. 2b). Differences in the number of CpG sites with different methylation patterns were most pronounced when filtered to include only imprinted loci (Fig. 2c, d). Methylation data (unmethylated + methylated)/(unmethylated + methylated + partially methylated) were normalized with tissue lines as the unmethylated baseline. Requiring partially methylated sites to be consistent across all five tissue samples yielded identification of 92 CgG sites representing 23 of 47 imprinted genes. When one tissue sample was allowed discordance (4/5 agreed), 30 of the 47 were identified. As expected of cells of uniparental origin, 49% (45 of 92) of CpG sites were either methylated or unmethylated and 100% of these sites were consistent with maternal imprinting. Probes identified as imprinted by partial methylation status in tissue samples were consistently partially methylated in the cell line samples as well. Of the 47 imprinted genes in beadstudio, 45 were differentially methylated between the phESC samples and hESC samples.

### NPC Differentiation

**Figure 3:**
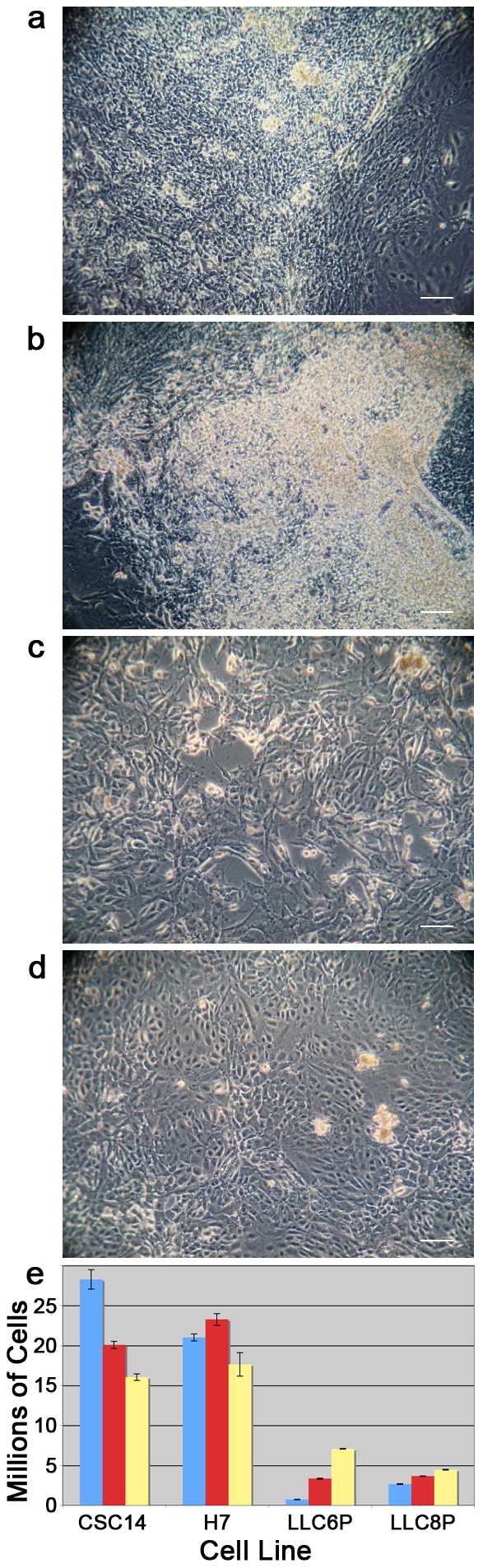
Exposure of hESC and phESC to a neural differentiation protocol yields quantitatively distinct cell populations. Conventionally-derived (a and b) and parthenote-derived (c and d) hESC have distinct growth patterns after the NPC differentiation protocol. Histograms plotting cellular yield (in millions of cells) after NPC differentiation (e) reveal that parthenote-derived lines yield fewer cells after differentiation than conventionally-derived lines. Scale bars represent 10 µm. Blue bars indicate differentiation 1, red bars indicate differentiation 2 and yellow bars indicate differentiation 3, representing the three successive differentiations.

On the last day of the standard differentiation protocol (Day 21), cells were plated at lower density for terminal differentiation. hESC-derived NPC cultures (Fig. 3a, b) were dense whereas phESC-derived cultures (Fig. 3c, d) were not. At no point do cells in this protocol form rosettes. Three successive differentiations of each line were quantified (Fig. 3e), each grow beginning as 75 cm^2^ original surface area of subconfluent hESC. The CSC14 line yielded 28.25 (±1.20), 20.06 (±0.45) and 16.03 (±0.41) million cells for a mean of 21.45 (±2.935) million. The H7 line yielded 21.00 (±0.44), 23.25 (±0.73) and 17.62 (±1.47) million cells for a mean of 20.62 (±1.336) million. The LLC6P line yielded 0.73 (±0.03), 3.34 (±0.07) and 7.06 (±0.03) million cells for a mean of 3.71 (±1.501) million. The LLC8P line yielded 2.65 (±0.04), 3.65 (±0.02) and 4.46 (±0.05) million cells for a mean of 3.59 (±0.427) million cells.

**Figure 4:**
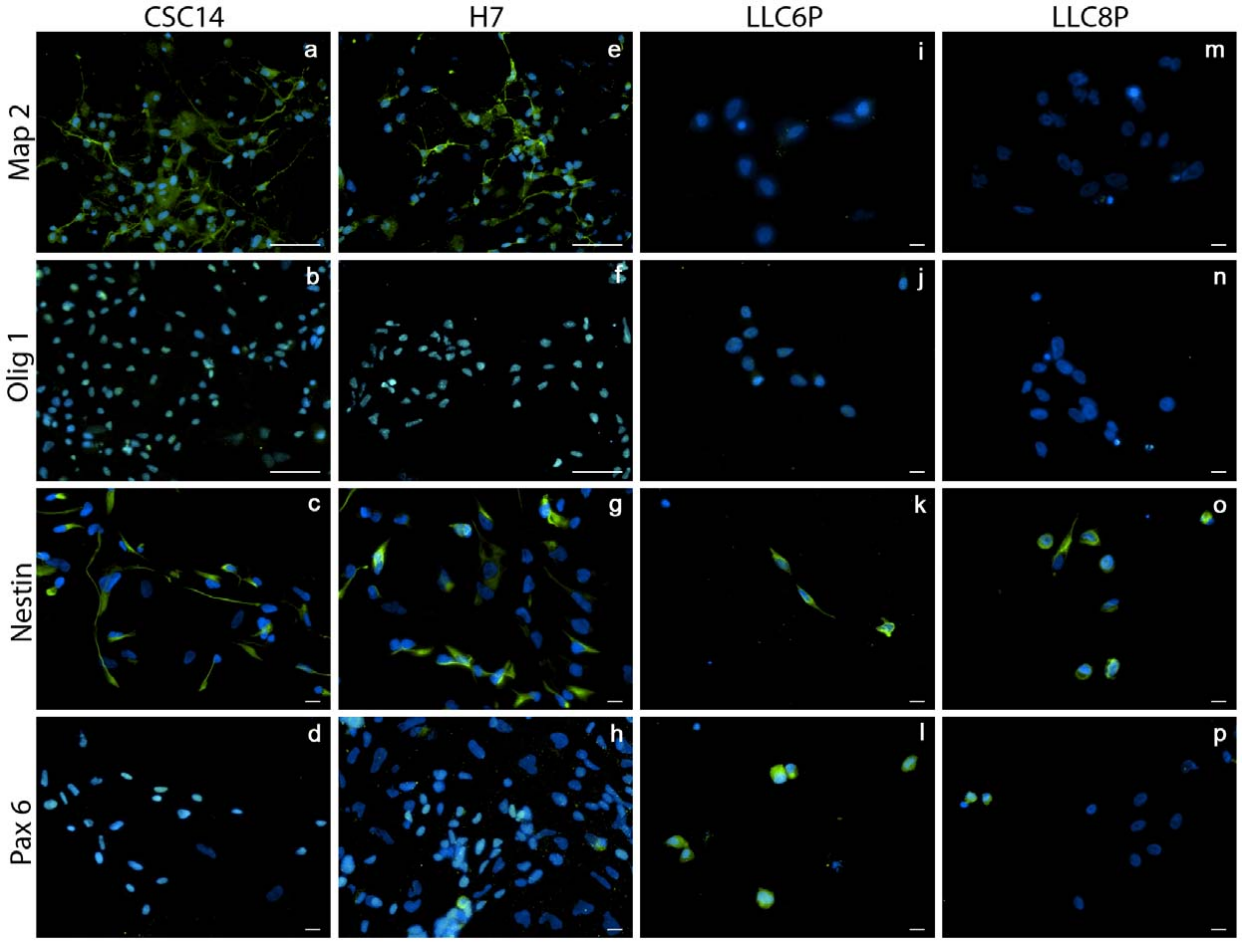
Immunocytochemical profile following NPC differentiation. Parthenote-derived lines displayed impaired neural maturation, but expressed immature markers normally. CSC14 (a–d), H7 (e–h), LLC6P (i–l) and LLC8P (m–p). Map2 (a, e, i and m), Olig1 (b, f, j and n), Nestin (c, g, k and o) and Pax6 (d, h, l and p). Scale bars represent 50 µm (a, b, e and f) and 10 µm (c, d, and g–p).

NPC were characterized on day 24 of culture (Fig. 4). Many NPC derivates of hESC origin labeled positive for Nestin, Pax6 and Map2 and virtually all labeled positive for Olig1, indicating a neuronal phenotype. NPC derivates of phESC origin did not label with Map2 and Olig1, but many labeled immunopositive for Nestin and Pax6, indicating a relatively immature neuronal phenotype. No SSEA 3/4-positive cells from the hESC groups could be detected, indicating that these cultures did not contain any undifferentiated stem cells. Occasional clusters of SSEA 3/4-positive cells were observed in the phESC group, indicating that these cells persisted in the undifferentiated state despite lineage direction provided by the differentiation protocol. Occasional single GFAP positive cells were observed in derivates of all four cell lines, indicating a low astrocyte presence within cultures. Cells showed no immunoreactivity in no-primary-antibody immunocytochemical control staining chambers.

**Figure 5:**
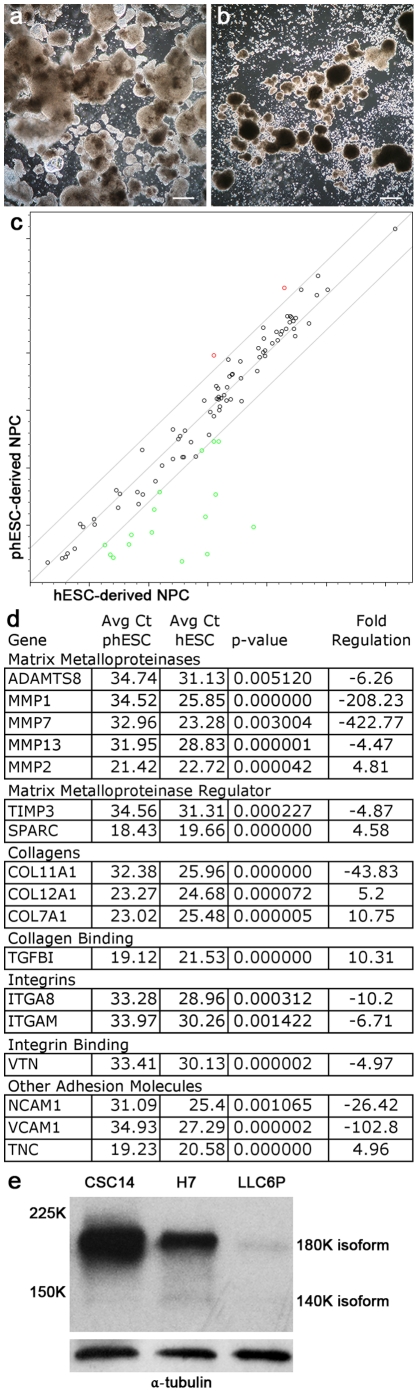
phESC display altered ECM profile and do not readily form spheres in non-adherent culture. During the sphere-forming stage of the NPC protocol, hESC gave rise to clusters that readily formed spheres with minimal dissociation (a). phESC at the same stage consistently dissociated (b). PCR arrays revealed differences in extracellular matrix and adhesion molecules in NPC (c). Parthenote-derived lines LLC6P and LLC8P are plotted on the y-axis and conventionally-derived lines H7 and CSC14 on the x-axis. Details of differentially expressed genes are presented numerically (d). Western blot analysis confirmed differential expression of NCAM1 (e). Scale bars represent 20 µm (a–b).

During the phase of the NPC protocol where cells are plated in ultra-low adherence 6-well plates, the phESC required particularly gentle feeding techniques as the spheres required to achieve appropriate cell-cell signaling were more prone to dissociation than those of their hESC counterparts (Fig. 5a, 5b).

Extracellular matrix expression as determined by ECM PCR array showed differences (Fig. 5c). Compared to hESC, phESC showed -422.77 fold-regulation of MMP7, -208.23 fold-regulation of MMP1, -102.8 fold-regulation of VCAM1 and -26.42 fold-regulation of NCAM1. Other differences are summarized in Fig. 5d. Western blot analysis confirmed differential expression of NCAM1 (Fig. 5e). The 180K isoform of NCAM1 commonly found in the central nervous system was clearly detected in hESC-derived NPC samples. Neither the 180K nor the 140K isoforms were detected in great amounts in phESC-derived NPC sample.

### RPE Differentiation

**Figure 6:**
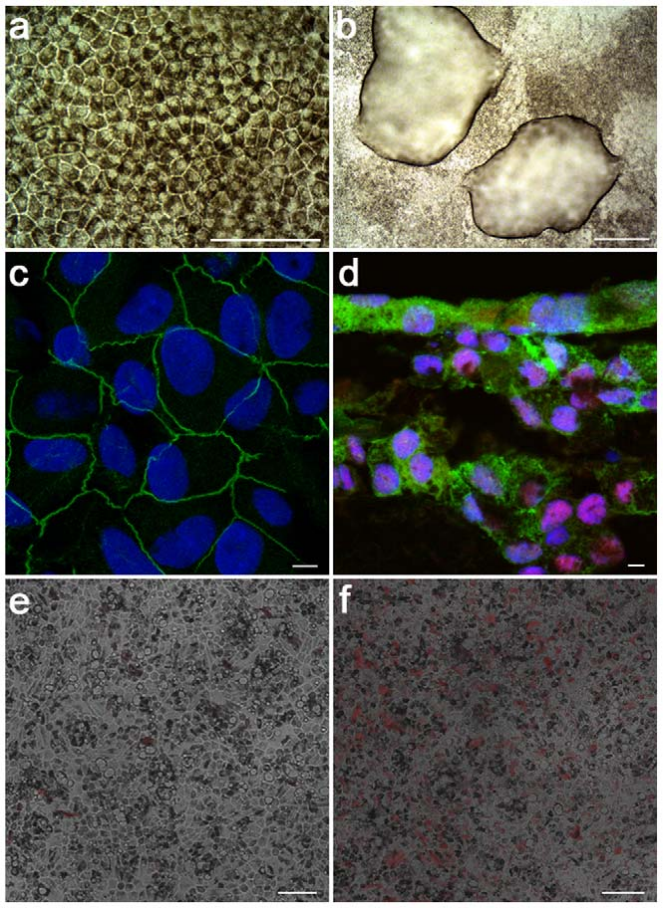
phESC produce functional RPE cells after differentiation. The LLC6P line produces pigmented cells in culture (a) and characteristic “dome-like” structures (b). Cells express RPE markers Occludin (c), CRALBP and mitf (d). Functionality of LLC6P-derived RPE is demonstrated by pHrhodo pH-sensitive fluorescent particle phagocytosis (e and d). Five minutes after administration of pHrhodo (e), RPE exhibit minimal vesicular pHrhodo content. Two hours and five minutes after administration (f), RPE exhibit marked increase in vesicular pHrhodo content. Scale bars represent 100 µm (a, e, f), 500 µm (b) and 5 µm (c, d).

When phESC and hESC cultures were allowed to spontaneously differentiate, both cell types lost their typical undifferentiated morphology and formed three-dimensional multicellular structures. Pigmented colonies of epithelial-like cells surrounded by cells of other phenotypes became visible approximately 4–6 weeks after bFGF withdrawal from culture medium; 8 weeks later a significant number of pigmented colonies were visible as “freckles” in the culture dishes of both tested cell types. Pigmented cells were handpicked and plated onto gelatin coated dishes for outgrowth. The cells lost pigmentation and epithelial morphology as they divided and migrated away from the initial attachment site, however, once confluence was established, the cells reverted to epithelial morphology and re-expressed pigment (Fig. 6a) as has been previously described for RPE [11], [12], [13] and for hESC-derived RPE [10]. These established monolayers of RPE-like cells were routinely passaged every 2–4 weeks and have undergone six passages to date. Cultures derived from both hESC and phESC demonstrated typical characteristics of RPE. During cultivation of established RPE monolayers “dome”-like structures were observed (Fig. 6b). The presence of these structures indicates proper polarization, formation of tight junctions and transport of ions to the basal side [14], [15].

The immunolocalization of RPE-specific markers demonstrated that both phESC- and hESC- derived RPE express Occludin (Fig. 6c), CRALBP, mitf (Fig. 6d), RPE65, Bestrophin, and tight junction marker ZO-1. The expression of RPE65 and Bestrophin were confirmed by western blot analysis (data not shown).

Exposure of differentiated cells to pHrhodo BioParticles demonstrated RPEs derived from phESC are capable of phagocytosis. Five minutes after application of the fluorescent particles, cells contained relatively little vesicular pHrhodo (Fig. 6e). Two hours later, vesicular pHrhodo content markedly increased (Fig. 6f). Confocal imaging after fixation confirmed that the fluorescent particles were contained within the cells (data not shown).

The findings outlined above were consistent using two distinct differentiation protocols, both of which lacked a sphere-forming stage. The first protocol was used to illustrate the principle that phESC were capable of end-product differentiation using protocols that lack a sphere-forming stage. The second protocol was used to confirm this finding.

## Discussion

Human embryonic stem cells can give rise to all cell types in the body and are a valuable tool to make differentiated cells for drug screening, predictive toxicology and potentially therapeutic applications. Therapeutic use of stem cell derived cell populations would be augmented if they did not induce an immune response in the host that would result in rejection of cells. Although preliminary data suggest that cell transplant populations differentiated from hESCs in vitro do not express an immunogenic HLA profile [16], the long-term immune effects are unknown. It is most likely that an autologous or immune-matched transplant would be best tolerated by the recipient. phESCs are pluripotent stem cells that can be made HLA homozygous for simplified immune matching, but their differentiation potential and biases are understudied. A greater understanding of the differentiation potential and differentiation bias of multiple cell types is needed to allow researchers to select the cell type that best suits the research or clinical need at hand. In this study we identify points of equivalence and non-equivalence between hESC and phESC.

Our data indicate that the in vitro directed NPC differentiation protocol resulted in poor yield from phESC compared to hESC, most likely due to a difference in cell-cell interaction resulting from differences in ECM expression. We corrected for the possibility that decreased proliferation in the undifferentiated state led to an apparent difference in final yield by maintaining undifferentiated cultures as long as was necessary to reach comparable confluence. The NPC protocol included a culture step consisting of neurosphere formation, which relied heavily on cell-cell interaction. Our PCR data revealed that phESCs and hESCs had a different expression pattern of extracellular matrix proteins, suggesting that while the neurosphere step may be ideal for the hESC cultures, it may be detrimental to the phESC cultures. Indeed, we observed a lack of adherence between phESC derivates, resulting in poor sphere forming capability, and poor adherence to surfaces. At day 6 through day 14 of the differentiation protocol, hESCs typically formed round, relatively stable spheres that held together during feeding of cells in suspension. phESCs had a tendency to dissociate and form smaller spheres or dissociate to the single-cell level. Since this feeding selects for the larger, uniform hESC-type spheres, it is not surprising that a problem at this stage would lead to dramatically decreased yield.

The possibility that phESCs cannot efficiently produce cells of the neural lineage is ruled out by the successful generation of RPE, which are of the neural lineage. The RPE yield and purity was equivalent in the phESC and hESC cultures. Notably, the RPE differentiation protocols did not rely on a sphere-formation step during culture, suggesting that the apparent inequality in differentiation potential demonstrated in the NPC differentiation study may have resulted from differences in ECM proteins and was thus an artifact of the protocol.

Our PCR data support the idea that the altered ECM profile of phESC derivates may contribute to their poor spherogenic nature. Multiple adhesion molecules and their regulators are expressed at different levels between hESC and phESC derivates. Relevant to successful generation of RPE from phESC, Col7A1, a collagen present in several layers of the retina [17], was elevated in phESC derivates. hESC derivates express elevated levels of four MMPs, indicating expression of these MMPs does not preclude sphere formation at this stage, possibly because co-expression of their regulators or targets ameliorate their anti-adhesive effects. For example, MMP13 (elevated in hESC derivates) preferentially cleaves type II collagen [18], which is also elevated in hESC as Col11A1. In addition, hESC derivates express elevated TIMP2, an inhibitor of MMPs [19]. hESC derivates express 26.42 fold higher levels of NCAM1 than their phESC counterparts. Decreased NCAM1 expression may contribute to loss of cells, both by direct dissociation leading to cell loss during aspiration of consumed media, and by loss of trophic support. NCAM1 activates the FGF receptor when clustered NCAM1 is involved in trans-homophilic binding [20]. FGF provides not only differentiation cues, but also trophic support [21], without which the phESC in our NPC protocol may have been at an additional disadvantage.

For therapeutic use, there are a number of practical considerations when choosing the appropriate cell type. Should their differentiation methods be optimized, parthenotes offer tremendous advantages for immune tolerance. Similar advantages could be realized with hESCs and iPS, however; there are drawbacks to using these two cell types that can be avoided with phESCs. hESCs express no human leukocyte antigen (HLA)-DR, DP, DQ and only low levels of HLA-A, B, C even when exposed to pro-inflammatory cytokines [16], [22], [23], but it is not yet clear how differentiated cells would be tolerated long-term. iPS technology suffers from increased tumorigenesis, low efficiency, and the time it takes to generate an immune-matched line would render it inadequate for acute needs. An additional commercial strength of phESCs is that their increased immune tolerance may broaden the range of patients who stand to benefit from each line. With rising health care costs, efficient production may be essential to opening the possibility of therapy to the greatest number of patients. A system in which a new line must be created for each patient may not be economically feasible, and will be technically inferior. phESCs can be immune matched for simplified long-term tolerance and can be banked and stored for efficient production and timely availability. When choosing a line for commercial scale up, cellular yield is an important measure to consider. The cost of expansion and differentiation can be immense, so addition of financial and temporal burdens caused by decreased yield is not desirable. These findings and needs underscore the importance of determining comparative differentiation biases and differentiation potential.

In conclusion, our data indicate that stem cell differentiation protocols are specific to stem cell types, and that phESCs have a unique ECM and methylation profile which renders differentiation protocols containing a spherogenic step unsuitable for this stem cell population. Thus, inappropriate differentiation protocols mask differentiation potential.
